# CT-based study on the safety margin of the anteromedial portal in elbow arthroscopy

**DOI:** 10.1038/s41598-025-21280-3

**Published:** 2025-10-07

**Authors:** Tobias Seidel, Sebastian Wegmann, Maximilian Weber, Tim Leschinger, Lars Peter Müller, Michael Hackl

**Affiliations:** 1 Clinic for Trauma Surgery, Orthopaedics, and Geriatric Traumatology, Evangelic Hospital Kalk, Buchforststr. 2, 51103 Cologne, Germany; 2 Department for Orthopaedics, Trauma Surgery and Plastic-Aesthetic Surgery, University Hospital Cologne, Kerpener Straße 62, 50937 Cologne, Germany

**Keywords:** Elbow, Median nerve, Arthroscopic anteromedial approach, Neurovascular, Complications, Ligaments, Trauma

## Abstract

With the increasing use of elbow arthroscopy, there is a growing concern about the risk of injury to neural structures, particularly the median nerve, when the anteromedial portal is created. This risk, which was previously underestimated, underscores the need for a guide to safe access that can significantly impact surgical practices and patient outcomes. We retrospectively evaluated 83 computer tomographies of the elbow without a higher grade of osteoarthritis and surgical treatment. In a 3D reconstruction, the median nerve was located, and a circle was set around it with a radius of 1.5 cm. This was supposed to represent a safety distance. Now, a tangent line was drawn from the tip of the processus coronoideus along the created circle, and the angle to the trans epicondylar plane was measured. The two legs of the angle crossed the skin. Therefore, the distance between these two intersections was also measured. Using IBM SPSS, we tested the data for normal distribution. The mean angle was 57.38°, and the mean distance between the skin intersections was 4.77 cm. The results suggest that the anteromedial portal, approximately 4.77 cm ventrally, measured from the medial epicondyle and at an angle of 57.38° in the trans epicondylar axis, can be suggested as a safe portal placed with reduced risk of damage to the median nerve based on imagining, with a safety distance of 1.5 cm. This can be helpful during elbow arthroscopy; clinical validation is yet to be performed.

## Introduction

A comprehensive understanding of anatomy is a prerequisite for successfully treating elbow pathologies. This is particularly crucial when creating the anteromedial portal during elbow arthroscopy, as it helps to minimize the risk of injury to neurovascular structures, especially the median nerve.

Only those who know the exact course of the neurovascular structures can minimize iatrogenic damage in surgical interventions.

### Neuroanatomie—N. medianus

**Fig. 1:**
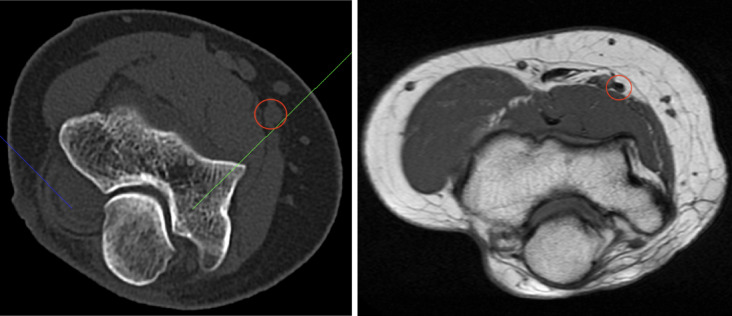
Illustration of the median nerve (red circle), CT on the left, MRI on the right.

The median nerve supplies most wrist and finger flexors, except the flexor carpi ulnaris, flexor digiti minimi brevis, and the ulnar half of the flexor digitorum profundus. It also provides sensory innervation to the radial palm, thenar region, radial 3$${\raise0.5ex\hbox{$\scriptstyle 1$} \kern-0.1em/\kern-0.15em \lower0.25ex\hbox{$\scriptstyle 2$}}$$ fingers (palmar side), and the fingertips of digits II–III and radial half of IV (dorsal side)^1^. Along this path, it releases its ramus musculares (see Fig. 1).

From the axilla, the nerve descends in the sulcus bicipitalis, usually ventral to the medial humeral condyle, though occasionally medial to the trochlea. Along this course, it gives off muscular branches. Distally, it passes between the two heads of the pronator teres, where the anterior interosseous nerve originates, then continues through the carpal tunnel to divide into palmar digital branches^2–4^.

Standardised portal placement at the elbow—in the context of neurovascular anatomy at the elbow.

Elbow joint arthroscopy is a commonly used therapy. It is technically complex and requires a high level of expertise from the surgeon. Several approaches have been recommended in numerous articles on this procedure^5–9^. Current recommendations for anteromedial portal placement in elbow arthroscopy are primarily derived from cadaveric dissections and surgical expertise. While these approaches provide valuable anatomical insights, they are limited by small sample sizes, altered tissue characteristics in cadaveric material, and variability in surgeon experience. As a result, precise quantitative data on neurovascular safety margins in living patients remain lacking. A CT-based approach offers a reproducible, imaging-derived method to define these relationships in a larger patient cohort, thereby addressing this gap and providing a more reliable foundation for safe portal placement.

Furthermore, several systematic reviews have investigated the complications following elbow arthroscopy. Tsenkov and Dimitrov assessed a rate for nerve injury of 1.26–7.5%, Klerk et al. recorded nerve palsies with 31% as the most frequent complication^10,11^.

**Fig. 2:**
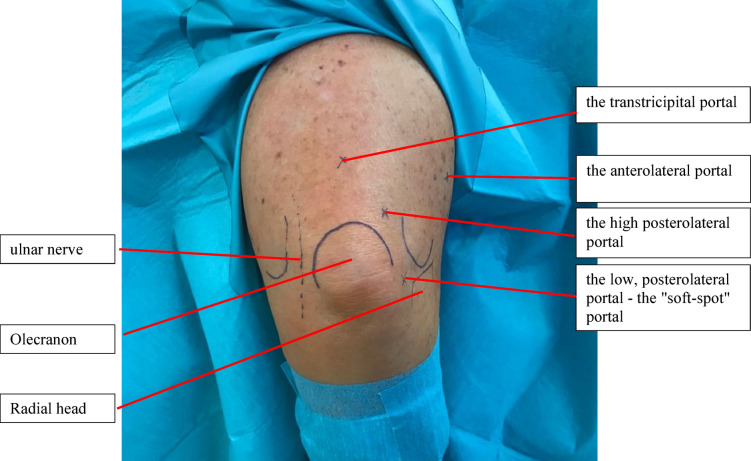
Arthroscopy portals of the elbow.

Elbow arthroscopy can be performed in the supine, abdominal, and lateral positions^7^. After the tourniquet has been applied, sterile dressings have been used, and the team has been fully time-out, the landmarks (epicondyles, olecranon tip, and ulnar nerve) can be marked with a sterile pen. Figure 2 represents an intraoperative representation of this step. The joint is filled with approximately 20 ml of sterile saline solution via the transtricipital portal. This allows easier access to the joint and increases the distance of the neurovascular bundles to the bony parts of the joint^12,13^.

### The anteromedial portal

An interchangeable rod is inserted via the anterolateral portal for arthroscopy of the ventral joint components. Then, the anteromedial portal is created on the medial side under camera control using the inside-out or outside-in technique. According to Andrews and Carson, this should be about 2 cm ventral and 2 cm distal to the medial epicondyle (approach)^14^. This portal provides ventral joint access, but the brachialis must be preserved to avoid neurovascular injury, Haapinemi et al. reported a severe case after arthroscopic arthrolysis with improved motion, yet a complete paresis of the median and radial nerve due to a discontinuity of 2.5–3 cm^15^.

By identifying a safe range in which median nerve injury would appear to be as minimal as possible with the anteromedial portal, our study has the potential to significantly reduce the risk of significant complications in elbow arthroscopy.

## Materials and methods

We retrospectively evaluated 83 computer tomographies of the elbow from January 1, 2015, to April 4, 2018. The study was conducted in accordance with the principles outlined in the Declaration of Helsinki, as revised in its most recent version. The study protocol was approved by the ethics committee of the University of Cologne. Based on their assessment, a formal ethics approval was not required.

An a priori power analysis indicated that, with a statistical power of 0.90 and a significance level of α = 0.05, a minimum sample size of 61 subjects would be required to achieve statistical significance.

CT scans were obtained with the forearm in neutral rotation (midway between pronation and supination) and the elbow positioned as close as possible to 90° of flexion to approximate the intraoperative setting. Scans exhibiting excessive flexion, hyperextension, or other extreme joint positions were excluded from the analysis. The 90° flexion position was chosen because it reduces neural tension and increases the distance between neurovascular structures and the joint, thereby reflecting the standard intraoperative safety position.

The glenohumeral joint was intact, without a higher grade (Kellgren and Lawrence Grade 0–2) of osteoarthritis or surgical treatment.

The following data were collected:

- Age
- Gender
- Side of the CT scan
- Angle between circle tangent and transepicodylar plane
- Distance of skin intersection between the two angle legs

Based on the CT data, three-dimensional reconstructions were generated to visualize the anatomical planes (sagittal, frontal, and transverse). To standardize orientation across all scans, the longitudinal axis of the humerus was aligned, defining both the frontal and sagittal planes. All subsequent measurements were then performed in the transverse plane.

Anatomical landmarks were established by first drawing the transepicondylar axis through the most distal points of the medial and lateral epicondyles. From this axis, a reference line was extended to the tip of the coronoid process at the distal margin of the trochlear notch (incisura semilunaris). This alignment ensured consistent and reproducible measurements between subjects, and the reference axes remained fixed throughout the procedure. The previously set axes remained unchanged during this procedure. Then, the median nerve was located, and a circle was set around it with a radius of 1.5 cm.

The median nerve was then identified on the CT reconstructions, and a circle with a radius of 1.5 cm was drawn around it. This radius was selected as a safety margin to account for anatomical variability and intraoperative factors (such as joint positioning or measurement inaccuracies) that could alter the relative location of the nerve. The 1.5 cm threshold is supported by previous cadaveric and imaging studies^16,17^. Drescher et al. demonstrated that when the elbow was flexed to 90° and the joint was distended, the median nerve lay on average 15.5 mm (range 8–27 mm) from the arthroscope introduced through the anteromedial portal^18^.

**Fig. 3:**
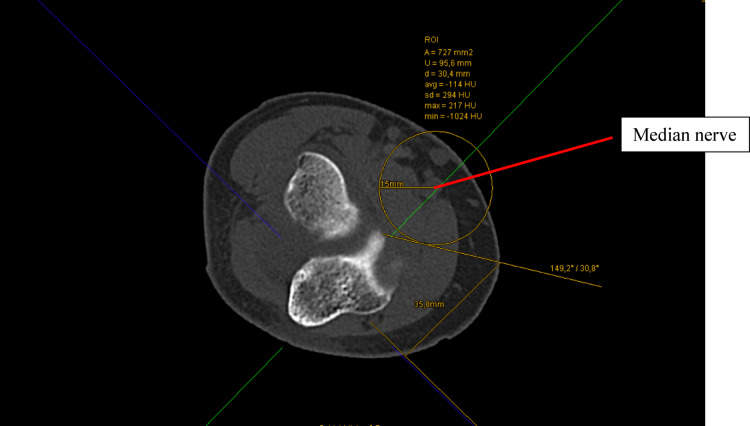
Measurement method in the transverse plane.

From the tip of the coronoid process, a tangent line was extended along the perimeter of this circle. The angle between this tangent and the transepicondylar axis was measured. Each leg of the angle intersected the skin surface, and the linear distance between these two points of intersection was also recorded (see Fig. 1). Figure 3 graphically displays the method used to measure the median nerve.

### Statistical analysis

The measurements were performed by an orthopedic and traumatology specialist. (TS) The analysis was performed using IBM SPSS to verify the results. Significance was defined as *p* < 0.05. First, the data were tested for normal distribution using the Shapiro–Wilk-test, and the following hypotheses were formulated:

- H0 hypothesis: data are typically distributed.
- H1 hypothesis: data are not standard distributed.

The test procedure also included the determination of the mean, minimum, maximum, and standard deviation.

## Results

There were 34 female and 49 male patients. Of the 83 elbows, 54 were right elbows, and 29 were left elbows.

**Fig. 4:**
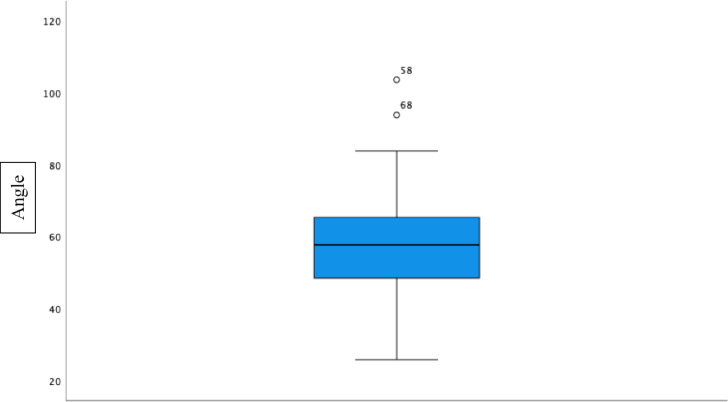
Boxplot diagram of the angle.

**Fig. 5:**
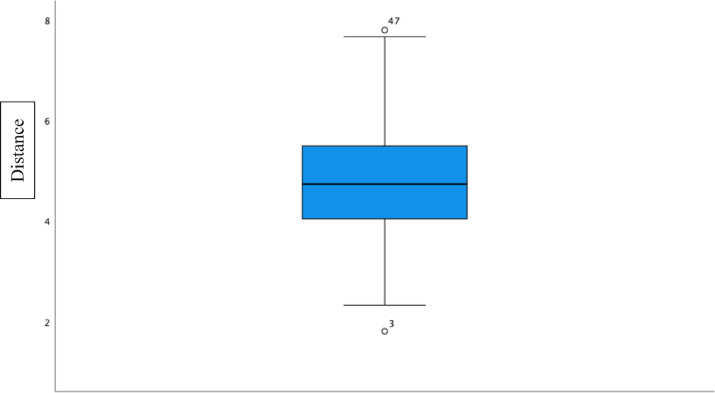
Boxplot diagram of the distance.

The mean value of the angle was 57.38°. The mean value of the distance between the intersections of the skin was 4.77 cm. The data were transferred to SPSS and tested for normal distribution. In the Shapiro–Wilk test, the significance of the angle was 0.476, and the importance of the distance was 0.346. The H0 hypothesis could not be rejected, and we have a normal distribution. To graphically display the results boxplot graphs were performed (Figs. 4 and 5). The results suggest that the anteromedial portal, approximately 4.77 cm ventrally, measured from the medial epicondyle and at an angle of 57.38° in the transepicondylar axis, can be placed with a good safety margin of 1.5 cm in order to prevent inducing damage to the median nerve.

## Discussion

The results of this study suggest that the anteromedial portal, placed approximately 4.77 cm ventrally (lower 95% CI: 4.53; Min: 1.81) from the medial epicondyle and at an angle of 57.38° (lower 95% CI: 54.29; Min: 25.80) in the trans epicondylar axis, can be placed without inducing damage to the median nerve, with a safety distance of 1.5 cm. The measurements and the results of the statistical analysis are represented in Table 1.

**Table 1:** Measurements and the results of the statistical analysis.

Investigation		Results angle (°)	Results distance (mm)
Mean value		57.38	4.77
95% confidence interval of the mean value	Lower limit	54.29	4.53
	Upper limit	60.48	5.02
Variance		205.95	1.25
Standard deviation		14.35	1.12
Minimum		25.80	1.81
Maximum		103.60	7.80

Elbow joint arthroscopy is a commonly used form of therapy, which, unfortunately, could be technically complex for the surgeon. Several systematic reviews have evaluated the risk of complications during elbow arthroscopy, and in general, they can be divided into preprocedural, peri- and post-procedural complications^11,19^.

In addition, there is a high risk of injury to the median nerve, especially during placement of the anteromedial portal and debridement of the ventral portions of the joint. One of the first reports of permanent iatrogenic nerve damage goes back to Thomas, Fast, and Shapiro^20^. The authors described a 20-year-old athlete who underwent elbow arthroscopy for an osteochondral lesion and was found postoperatively to have complete paresis of the profundus branch of the radial nerve. The conclusion was that there was a high risk of injury to nervous structures due to the anatomical location. Particular attention should be given to minimum values and outliers, as these represent critical scenarios in which the safety margin is reduced. Such cases are especially relevant in clinical practice, since even isolated deviations may significantly impact the risk of nerve injury for individual patients. Although the mean values and confidence intervals suggest that an anteromedial portal can be placed with a sufficient safety margin in most cases, the presence of outliers is particularly clinically relevant. These extreme values suggest that in some individuals, the neurovascular structures may lie considerably closer to the intended portal than the average would indicate. In practice, this variability means that relying solely on mean values could underestimate the risk of iatrogenic nerve injury in patients at the lower end of the distribution. From a clinical perspective, surgeons must therefore remain aware that a “safe” portal placement cannot be universally guaranteed, and additional safety measures—such as meticulous palpation of landmarks, joint insufflation, use of a proximal rather than distal portal, and real-time intraoperative assessment—should always be employed. Outliers underscore the importance of individualised evaluation rather than a one-size-fits-all approach.

Hackl et al. measured the distance from the median nerve to the trochlea and the medial border of the trochlea in 22 formalin-fixed upper extremities^1^. The distance from the median nerve to the trochlea was 11.7 mm (± 5.2 mm), and the distance to the medial border was 2.4 mm (± 4.1 mm). Although the safety margin is limited, several technical measures have been described to improve outcomes and minimize the risk of iatrogenic nerve injury. For instance, joint distension with 15–20 ml of fluid via the radial soft spot has been shown to increase the distance between the radial nerve and the underlying bone from approximately 4–11 mm. Furthermore, maintaining the elbow in 90° flexion during arthroscopy reduces neural tension and, once the joint is insufflated, further increases the separation of the neurovascular structures from the bony surface^21^.

Unexpectedly, the anteromedial portal is often described as a low-risk approach^17^. Making a difference between the proximal anteromedial portal and the distal anteromedial portal is necessary. According to Andrews and Carson, the proximal anteromedial portal (the original portal) is located 2 cm distal and 2 cm ventral to the medial epicondyle. Lindenfeld first defined the proximal anteromedial portal with an approach of 1 cm proximal and 1 cm ventral to the medial epicondyle^22^. According to a comparative study, the distance of the median nerve is 5.07 mm from the proximal portal. In these studies, the median nerve (18.05 ± 3.67 mm) had the longest distance from the proximal anteromedial portal^17^. The results demonstrate that the proximal anteromedial portal presents a lower risk for structures than the distal portal. With 90° flexion of the elbow, there is a lower risk of neurovascular injury during portal placement. These results suggest that the proximal anteromedial portal is safer for anteromedial elbow arthroscopy. Optimised portal placement minimises the risk of neurovascular injury.

Drescher et al. evaluated the distance of the median nerve from the usual Andrews approach in extension and 90° flexion, as well as in pro- and supination^18^. With the anteromedial approach, the mean distance of the median nerve from the arthroscope was 15.5 mm (± 8 to 27 mm) when the best joint position was used (90 degrees flexed joint, maximum supination of the forearm).

Thus, injury to the median nerve is not unrealistic and warrants higher attention.

The mean angle between the epicondyle lateralis and the established safety distance in our study was 57.38°. The mean value of the distance between the skin intersections was 4.77 cm. We consider it possible to place an arthroscopic portal from the epicondylus laterales about 4.77 cm in the direction of the Processus coronoideus without assuming relevant injury to the median nerve. In this case, the safety distance is 1.5 cm.

In the Shapiro–Wilk test, the significance of the angle was 0.476, and the importance of the distance was 0.346. Thus, the H0 hypothesis, which suggests a normal distribution, could not be rejected in both studies.

Quantile–quantile diagrams were created to verify the results. These diagrams show a normal distribution of the data around the straight line without a higher degree of variation, which supports the intended normal distribution of the data.

## Conclusions

The results suggest that the anteromedial portal, approximately 4.77 cm ventrally, measured from the medial epicondyle and at an angle of 57.38° in the trans epicondylar axis, can be suggested as a safe portal placed with reduced risk of damage to the median nerve based on imagining, with a safety distance of 1.5 cm.

This research is a theoretical translation of the actual situation, and therefore, further confirmatory studies are needed to verify the hypothesis and its applicability and adaptation in clinical practice. However, it provides a framework for further research of the neurovascular situation at the elbow joint in relation to the anteromedial portal. The authors must conclude that the anteromedial portal can be deemed a safe portal in this theoretical framework.

### Limitation of authorship

The use of CT scans for this study was based on the fact, that clinical applicability is higher, as CT scans are more often used and the higher availability in pathological situations.

Forearm position (pronation–supination) was generally neutral during CT scanning, although minor variations may have occurred. Such variations can influence measured elbow distances by reducing or increasing them. Further studies should investigate their influence on the nerve’s position.

The initially described access is 2 cm distal to the medial epicondyle. We have used the tip of the coronoid process as a reference as it is a good, palpable reference. Thus, the results can be better transferred to the operative situation. However, the exact distance of the coronoid process from the medial epicondyle is highly variable. Therefore, the measured level in the CT is also subject to a specific variability.

Besides the median nerve, the radial nerve and ulnar nerve are also at risk of being injured during elbow arthroscopy. Further studies, especially in vivo or cadaveric studies, should investigate all nerves in more detail.

Patient-related variables, such as BMI or muscle mass, and subgroup testing were not evaluated during this study and could influence the nerve’s position.

## Data Availability

The datasets generated and analysed during the current study are available from the corresponding author on reasonable request.
